# Synthesis, crystal structure and Hirshfeld surface analysis of 2-oxo-*N*-(pyridin-4-yl)-2*H*-chromene-3-carboxamide

**DOI:** 10.1107/S2056989026003798

**Published:** 2026-04-29

**Authors:** M. Sunithakumari, M. Harish Kumar, H. C. Devarajegowda, M. U. Gagan, V. Dwarakanath, B. S. Palakshamurthy

**Affiliations:** a Department of Physics Yuvaraja's College University of Mysore,Mysore-570005 Karnataka India; b Department of Biotechnology, UCS Tumkur University Tumkur Karnataka-572103 India; c Department of PG Studies and Research in Physics Albert Einstein Block UCS Tumkur University, Tumkur Karnataka-572103 India; Katholieke Universiteit Leuven, Belgium

**Keywords:** crystal structure, Hirshfeld surface, two-dimensional fingerprint, 2*H*-chromene-3-carboxamide

## Abstract

In the title compound, C_15_H_10_N_2_O_3_, the dihedral angle between the heterocyclic 2*H*-chromene and the pyridine moieties is 2.09 (9)°. The mol­ecule exhibits an almost planar conformation (r.m.s. deviation = 0.048 Å). The torsion angle associated with the amide linkage between the 2*H*-chromene and pyridine moieties is −179.17 (18)^ο^ and found to be a*nti-*periplanar. In the crystal, C—H⋯O hydrogen bonds form two sets of inversion dimers, generating *R*_2_^2^(14) and *R*_2_^2^(16) synthons that propagate along the [010] direction. The mol­ecular packing in the crystal is further consolidated by C= O⋯π inter­actions and π–π stacking with centroid–centroid separations of 3.732 (2), 3.815 (2) and 3.947 (2) Å. The two-dimensional fingerprint plots indicate that the major contributions to the crystal packing arise from H⋯O/O⋯H (19.5%), H⋯C/C⋯H (14.1%), H⋯N/N⋯H (8.0%) and O⋯C/C⋯O (5.0%) inter­actions.

## Abstract 2

In the crystal of the title compound, the mol­ecules are linked *via* C—H⋯O hydrogen bonds, giving rise to centrosymmetric inversion dimers. On one side of the mol­ecule, C—H⋯O inter­actions generate *R*^2^_2_(16)ring motifs, while on the opposite side, C—H⋯O hydrogen bonds form *R*^2^_2_(14) ring motifs.

## 1. Chemical context

The 2-oxo-2*H*-chromene (coumarin) scaffold is of considerable inter­est due to its diverse biological and physicochemical properties. Coumarin derivatives, both natural and synthetic, exhibit a broad range of pharmacological activities, including anti­oxidant, anti-inflammatory and anti­coagulant effects (Annunziata *et al.*, 2020 ▸; Ivanov *et al.*, 2025 ▸). They have also been reported to show significant anti­cancer activity through inhibition of cell proliferation and modulation of signalling pathways (Emami & Dadashpour, 2015 ▸), as well as anti­microbial activity against pathogens such as *Staphylococcus aureus* and *Escherichia coli* (Tang *et al.*, 2025 ▸; Baaiu *et al.*, 2025 ▸). In addition to pharmaceutical relevance, coumarin derivatives find applications in other fields owing to their characteristic odour, fluorescence properties and agrochemical activity (Anywar & Muhumuza, 2024 ▸). In particular, chromene-3-carboxamide derivatives have attracted attention due to their cytotoxic activity and their ability to inhibit cancer-related enzymes such as carbonic anhydrase IX (Supuran *et al.*, 2017 ▸). These compounds also exhibit anti­bacterial activity against both Gram-positive and Gram-negative bacteria (Khan *et al.*, 2020 ▸).

Furthermore, the presence of amide and heterocyclic donor groups, such as pyridin-4-yl moieties, enhances their ability to coordinate with metal ions, making them useful in coordination chemistry and crystal engineering (El-Sayed *et al.*, 2023 ▸; Kaur *et al.*, 2015 ▸). Such coordination behaviour facilitates the formation of supra­molecular assemblies and functional materials. On the basis of these considerations, coumarin–pyridine hybrid systems are of particular inter­est, and we report herein the synthesis and crystal structure of a new derivative.
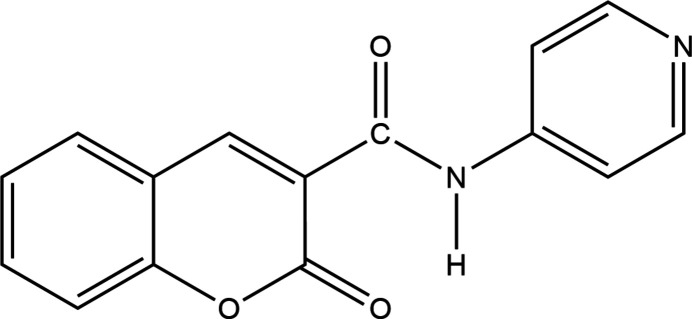


## 2. Structural commentary

**Figure 1:**
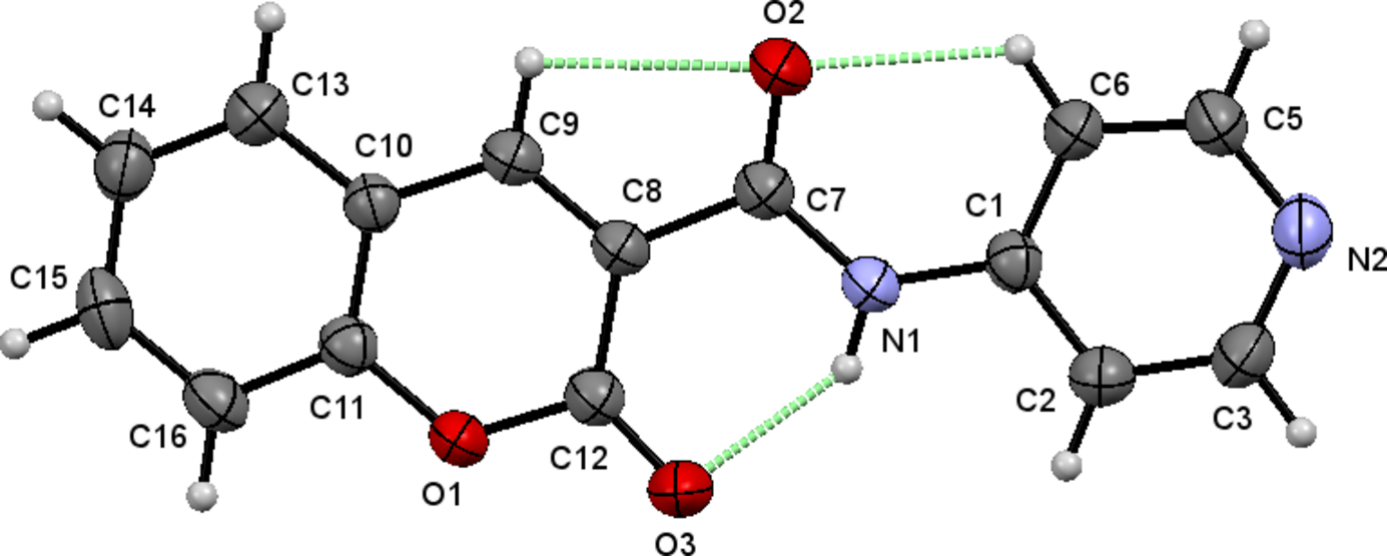
The title compound with the atom-numbering scheme and 50% probability ellipsoids.

In the title compound (Fig. 1 ▸), the fused 2*H*-chromene ring system (C8–C11/O1/C12–C16) and the pyridine ring (C1–C3/N2/C5/C6) are nearly coplanar, forming a dihedral angle of 2.09 (9)°, with an r.m.s. deviation of 0.048 Å, indicating an essentially planar conformation. The amide linkage between the 2*H*-chromene and pyridine moieties adopts an anti-periplanar conformation, as evidenced by the C8—C7(=O2)—N1—C1 torsion angle of −179.17 (18)°. Bond lengths and angles are within normal ranges. Intra­molecular N1—H1⋯O3, C6—H6⋯O2 and C9—H9⋯O2 hydrogen bonds are observed (see Table 1 ▸ for details), contributing to the stabilization of the mol­ecular conformation.

## 3. Supra­molecular features

**Figure 2:**
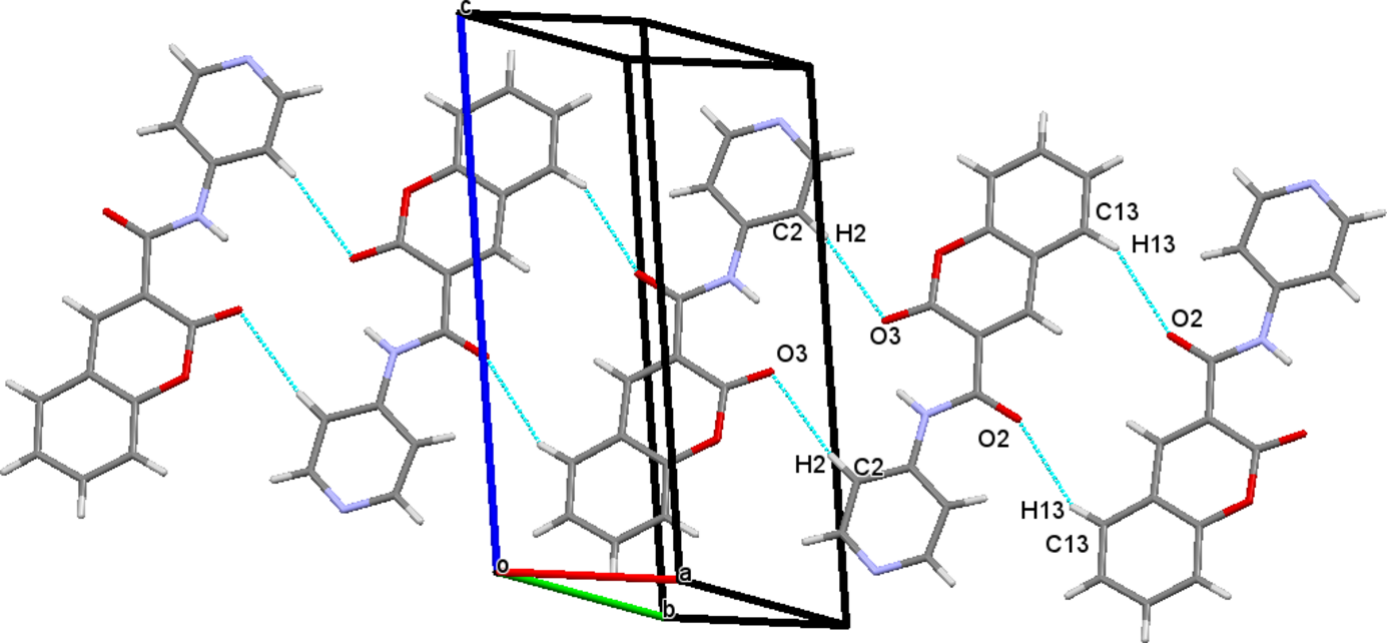
The packing of the title compound. Inter­molecular C—H⋯O hydrogen bonds are shown as blue dashed lines.

**Figure 3:**
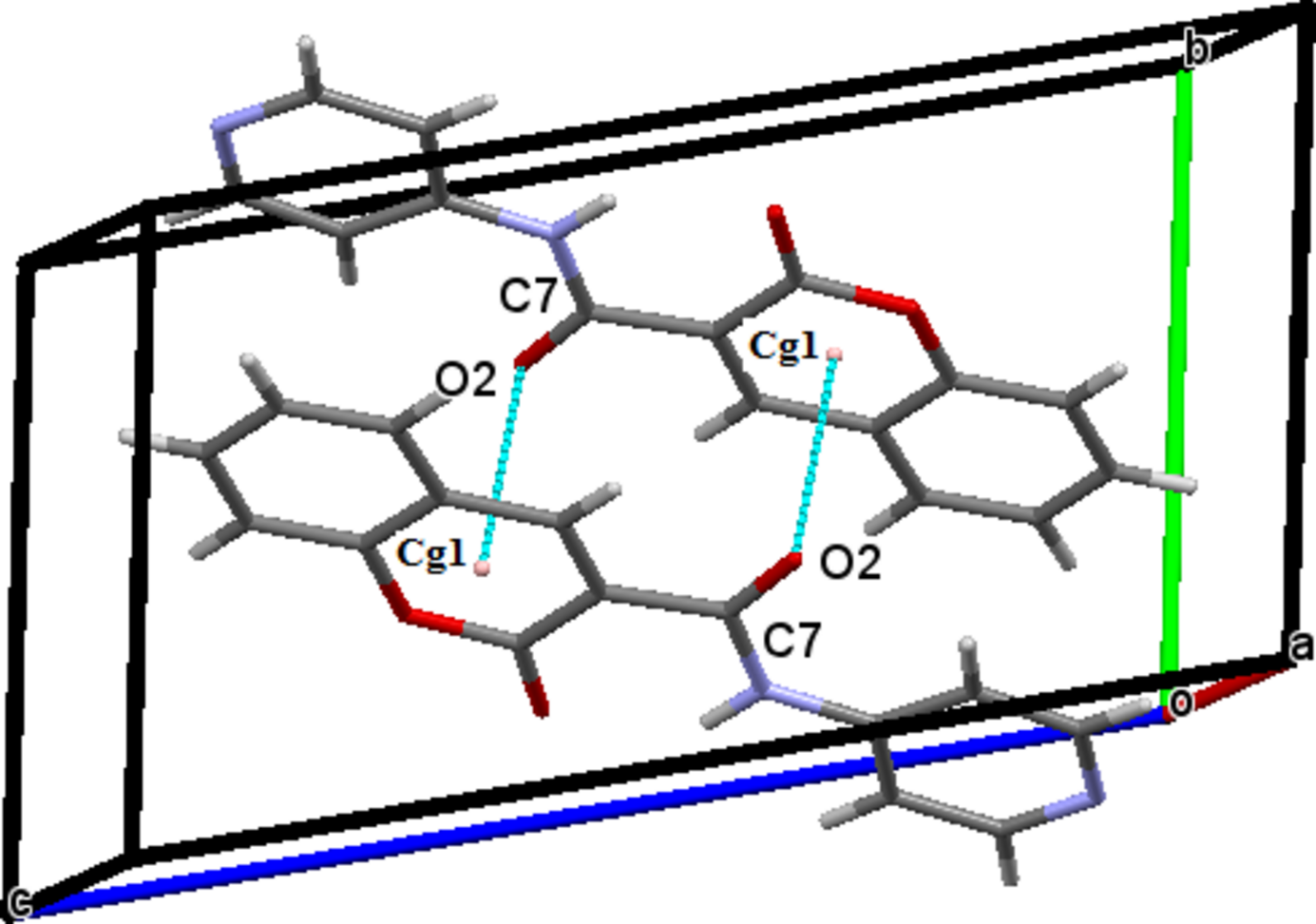
Partial crystal packing of the title compound showing the C—O⋯π inter­actions as blue dashed lines.

**Figure 4:**
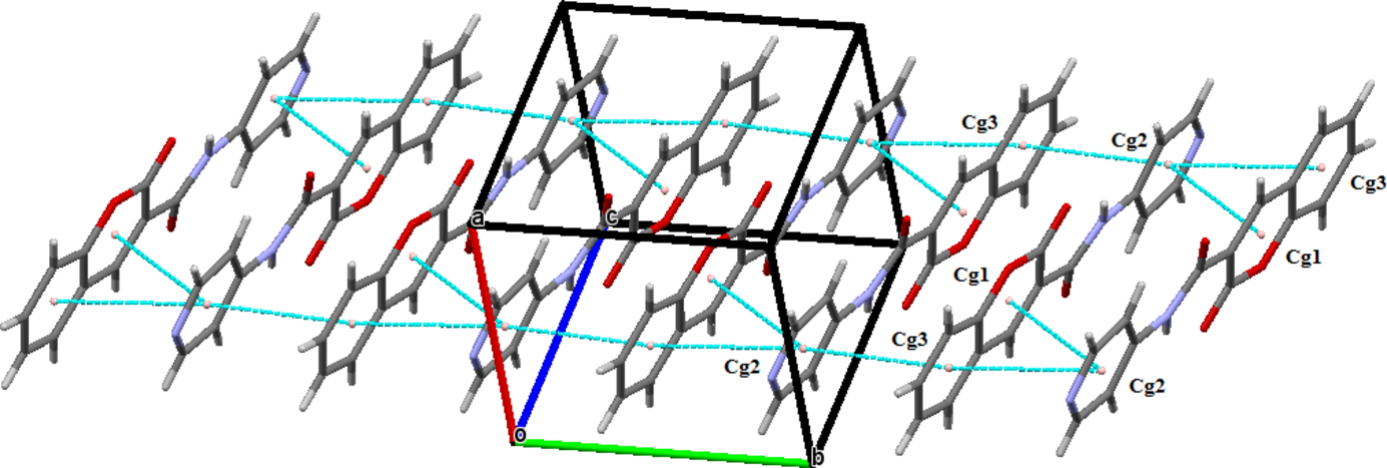
Crystal packing of the title compound showing the π–π inter­actions as blue dashed lines.

In the crystal, the mol­ecules are linked via C—H⋯O hydrogen bonds, giving rise to centrosymmetric inversion dimers (Table 1 ▸). On one side of the mol­ecule, C2—H2⋯O3 inter­actions generate 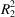
(16) ring motifs, while on the opposite side, C13—H13⋯O2 hydrogen bonds form 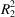
(14) ring motifs. These discrete supra­molecular synthons propagate along the [010] direction (Fig. 2 ▸). The crystal packing is further consolidated by C=O⋯π inter­actions involving the carbonyl group C7=O2 and the π-system of the 2*H*-chromene ring [Fig. 3 ▸; O2⋯*Cg*1(1 − *x*, 1 − *y*, 1 − *z*) = 3.279 (2) Å, *Cg*1 is the centroid of the O1/C8–C12 ring]. In addition, significant π–π stacking inter­actions are observed between adjacent aromatic systems (Fig. 4 ▸). The centroid–centroid separations are *Cg*1⋯*Cg*2(1 − *x*, −*y*, 1 − *z*) = 3.815 (2) Å, *Cg*2⋯*Cg*3(1 − *x*, 1 − *y*, 1 − *z*) = 3.732 (2) Å and *Cg*2⋯*Cg*3(1 − *x*, 2 − *y*, 1 − *z*) = 3.947 (2) Å, where *Cg*2 and *Cg*3 are the centroids of the pyridine (N2/C1–C3/C5–C6) and 2*H*-chromene benzene (C10/C11/C13–C16) rings, respectively. These non-covalent inter­actions collectively contribute to the cohesion of the three-dimensional crystal architecture.

## 4. Database survey

A search of the Cambridge Structural Database (CSD, version 5.43, 2025 update; Groom *et al.*, 2016 ▸) for structures containing the 2-oxo-2*H*-chromene-3-carboxamide moiety yielded more than 30 hits. Among these, closely related structures include 7-(di­ethyl­amino)-*N*-(4-nitro­phen­yl)-2-oxo-2*H*-chromene-3-carboxamide (refcode DISYIP; Maldonado-Domínguez *et al.*, 2014 ▸), 7-(di­ethyl­amino)-*N*-(4-fluoro­phen­yl)-2-oxo-2*H*-chromene-3-carboxamide (DISXUA; Maldonado-Domínguez *et al.*, 2014 ▸), and 7-(di­ethyl­amino)-*N*-(3-methyl-1,3-benzo­thia­zol-2(3*H*)-yl­idene)-2-oxo-2*H*-chromene-3-carboxamide (DUBHOZ; Wang *et al.*, 2015 ▸), which exhibit dihedral angles of 0.28, 0.79 and 2.85°, respectively, indicating a strong tendency toward planarity in this class of compounds similar to the title mol­ecule.

The torsional angle associated with the amide linkage in representative examples *N*-(3-(imidazo[1,2-*a*]pyridin-2-yl)phen­yl)-8-meth­oxy-2-oxo-2*H*-chromene-3-carboxamide (BONKAS; Julien *et al.*, 2014 ▸), 7-(di­ethyl­amino)-*N*-(4-fluoro­phen­yl)-2-oxo-2*H*-chromene-3-carboxamide, *N*-(4-cyano­phen­yl)-7-(di­ethyl­amino)-2-oxo-2*H*-chromene-3-carb­ox­amide and 7-(di­ethyl­amino)-*N*-(4-nitro­phen­yl)-2-oxo-2*H*-chromene-3-carboxamide (DISXUA, DISYEL and DISYIP, respectively; Maldonado-Domínguez *et al.*, 2014 ▸) are 179.96, 177.26, −179.17 and −177.43°, respectively, reflecting an anti-periplanar conformation. In the representative examples 7-(di­ethyl­amino)-*N*-(3-methyl-1,3-benzo­thia­zol-2(3*H*)-yl­idene)-2-oxo-2*H*-chromene-3-carboxamide (DUBHOZ; Wang *et al.*, 2015 ▸) and 6-meth­oxy-*N*-(3-methyl­phen­yl)-2-oxo-2*H*-chromene-3-carboxamide (ECEBIA; Gomes *et al.*, 2016 ▸) the torsion angles are −177.73 and −179.06°, respectively, reflecting the same anti-periplanar conformation as comparable to the title mol­ecule.

## 5. Hirshfeld surface analysis

**Figure 5:**
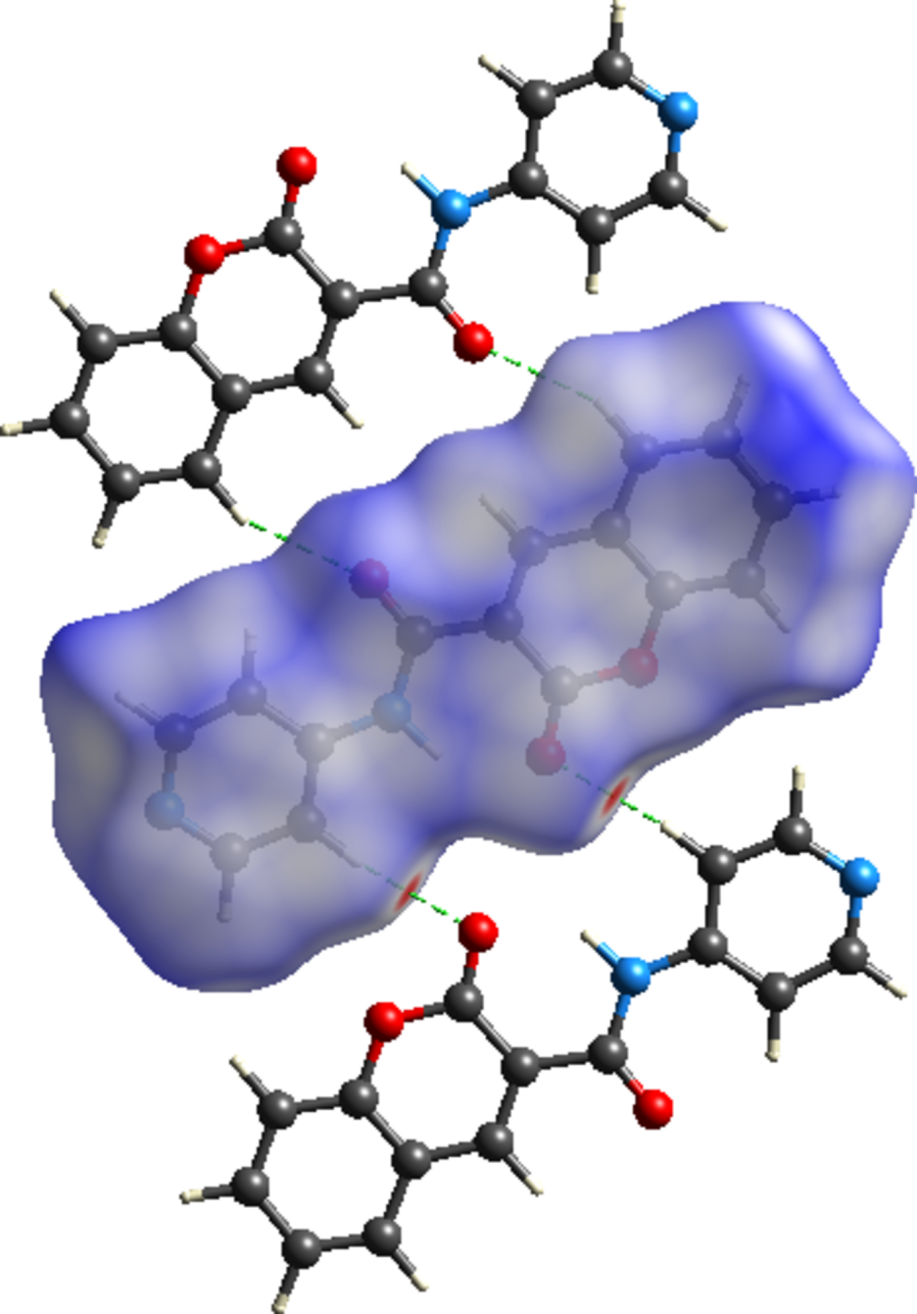
View of the three-dimensional Hirshfeld surface mapped over *d*_norm_ and the C—H⋯O inter­actions forming 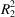
(16) and 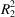
(14) ring motifs with neighboring mol­ecules

**Figure 6:**
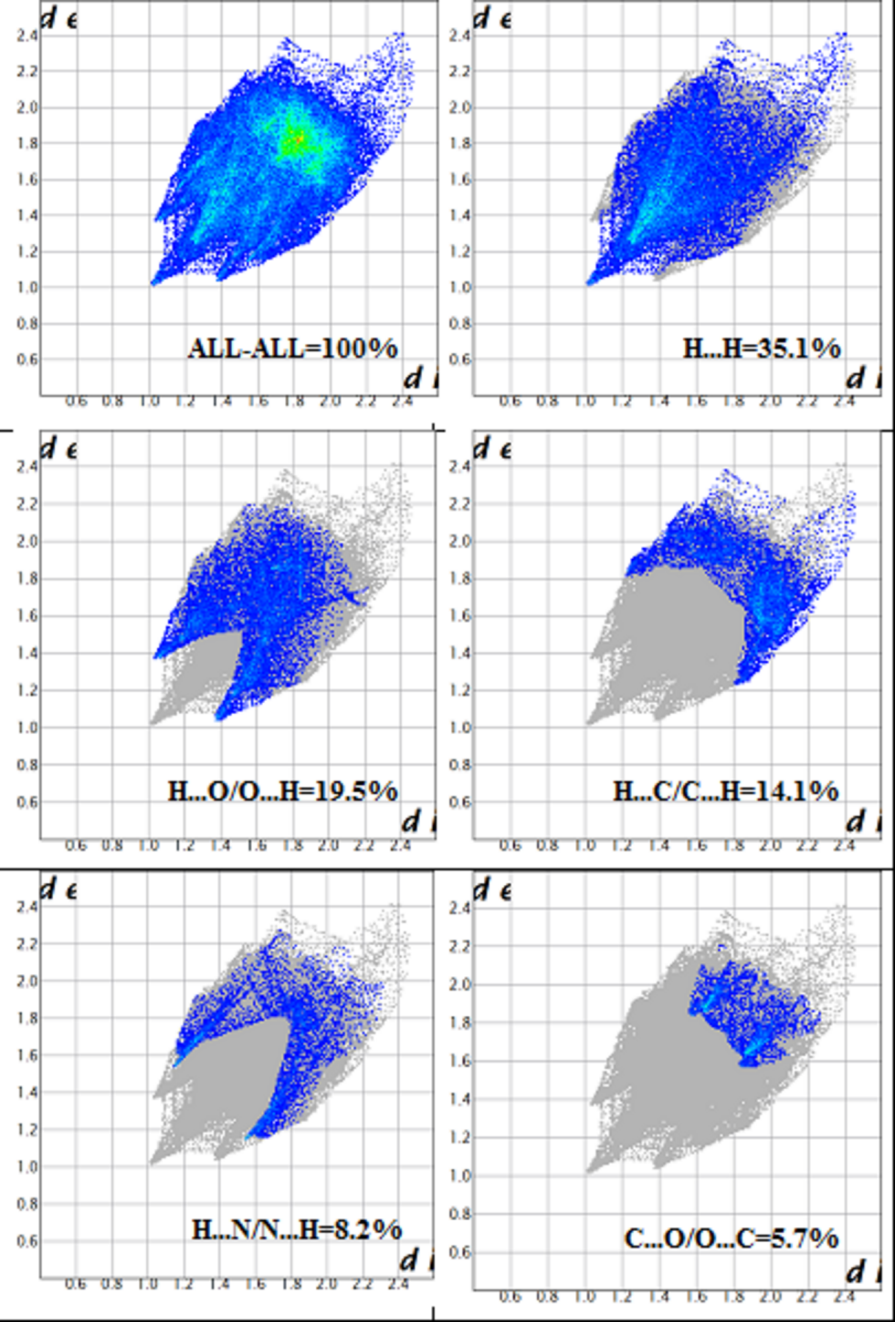
The two-dimensional fingerprint plots showing all (100%), H⋯H (31.5%), H⋯O/O⋯H (19.5%), H⋯C/C⋯H (14.1%), H⋯N/N⋯H (8.0%), and O⋯C/C⋯O (5.0%) contacts.

A Hirshfeld surface analysis of the title compound was carried out using *CrystalExplorer* (Spackman *et al.*, 2021 ▸) to investigate and visualize the inter­molecular inter­actions governing the crystal packing. The Hirshfeld surface mapped over *d*_norm_ together with two neighbouring mol­ecules is shown in Fig. 5 ▸, On one side of the mol­ecule, the oxygen atom of the amide linkage takes part in C2—H2⋯O3 inter­actions generating 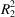
(16) ring motifs, while on the opposite side, the oxygen of the 2-oxo-2*H*-chromene moiety takes part in C13—H13⋯O2 hydrogen bonds forming 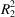
(14) ring motifs, where red spots correspond to regions of these close inter­molecular contacts. The two-dimensional fingerprint plots provide a qu­anti­tative description of the various inter­molecular inter­actions. The most significant contributions arise from H⋯H contacts (35.1%), followed by H⋯O/O⋯H (19.5%), H⋯C/C⋯H (14.1%), H⋯N/N⋯H (8.2%) and O⋯C/C⋯O (5.7%) inter­actions (Fig. 6 ▸). These results indicate that van der Waals inter­actions (H⋯H) dominate the crystal packing, while directional hydrogen-bonding and heteroatom contacts make notable secondary contributions.

## 6. Synthesis and crystallization

**Figure 7:**
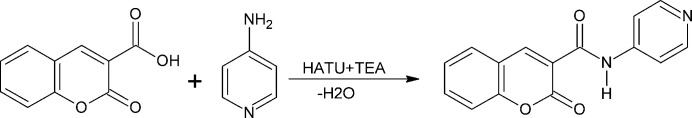
Reaction scheme for the synthesis of the title compound.

A mixture of 2-oxo-2*H*-chromene-3-carb­oxy­lic acid (1.00 mmol), 4-amino­pyridine (2.00 mmol) and triethyl amine (TEA; 4.2 mmol) in aceto­nitrile (15 ml) was stirred at room temperature for 15 min. Then, 1-[bis­(di­methyl­amino)­methyl­ene]-1*H*-1,2,3-triazolo[4,5-*b*]pyridinium 3-oxide hexa­fluoro­phosphate (HATU; 5 mmol) was added in one portion, and the reaction was covered with a rubber septum. After 24 h, the aceto­nitrile was removed *in vacuo*, and the residue was dissolved in di­chloro­methane (25 ml). The organic layer was washed with water (25 ml) and separated; the aqueous layer was extracted with di­chloro­methane. The combined organic layers were washed with brine, dried over magnesium sulfate, and concentrated under reduced pressure. The crude residue was purified by 60–120 mesh silica gel column chromatography (1:4 ethyl acetate:hexa­ne). The scheme for the reaction is presented in Fig. 7 ▸.

## 7. Refinement

Crystal data, data collection and structure refinement details are summarized in Table 2 ▸. All H atoms were positioned with idealized geometry (N—H = 0.83 Å, C—H = 0.93 Å) and refined using a riding model with *U*_iso_(H) = 1.2*U*_eq_(C/N).

## Supplementary Material

Crystal structure: contains datablock(s) I. DOI: 10.1107/S2056989026003798/vm2329sup1.cif

Structure factors: contains datablock(s) I. DOI: 10.1107/S2056989026003798/vm2329Isup3.hkl

Supporting information file. DOI: 10.1107/S2056989026003798/vm2329Isup3.cml

CCDC reference: 2545176

Additional supporting information:  crystallographic information; 3D view; checkCIF report

## Figures and Tables

**Table 1:** Hydrogen-bond geometry (Å, °)

*D*—H⋯*A*	*D*—H	H⋯*A*	*D*⋯*A*	*D*—H⋯*A*
N1—H1⋯O3	0.86	1.97	2.698 (2)	141
C6—H6⋯O2	0.93	2.29	2.869 (3)	120
C9—H9⋯O2	0.93	2.44	2.758 (3)	100
C2—H2⋯O3^i^	0.93	2.58	3.480 (3)	162
C13—H13⋯O2^ii^	0.93	2.59	3.422 (3)	149

Symmetry codes: (i) 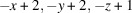; (ii) 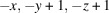.

**Table 2:** Experimental details

Crystal data	
Chemical formula	C_15_H_10_N_2_O_3_
*M*_r_	266.25
Crystal system, space group	Triclinic, *P* 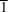
Temperature (K)	299
*a*, *b*, *c* (Å)	5.731 (2), 7.668 (3), 13.911 (5)
α, β, γ (°)	103.199 (14), 93.059 (11), 94.332 (10)
*V* (Å^3^)	591.8 (4)
*Z*	2
Radiation type	Mo *K*α
μ (mm^−1^)	0.11
Crystal size (mm)	0.32 × 0.27 × 0.24

Data collection	
Diffractometer	Bruker SMART APEXII CCD
Absorption correction	Multi-scan (*SADABS*; Krause *et al.*, 2015 ▸)
*T*_min_, *T*_max_	0.964, 0.973
No. of measured, independent and observed [*I* > 2σ(*I*)] reflections	8155, 3181, 2263
*R*_int_	0.034
(sin θ/λ)_max_ (Å^−1^)	0.697

Refinement	
*R*[*F*^2^ > 2σ(*F*^2^)], *wR*(*F*^2^), *S*	0.071, 0.184, 1.02
No. of reflections	3181
No. of parameters	181
H-atom treatment	H-atom parameters constrained
Δρ_max_, Δρ_min_ (e Å^−3^)	0.35, −0.22

Computer programs: *APEX2* and *SAINT* (Bruker, 2017 ▸), *SHELXT2018/3* (Sheldrick, 2015*a* ▸), *SHELXL2019/3* (Sheldrick, 2015*b* ▸), *Mercury* (Macrae *et al.*, 2020 ▸) and *publCIF* (Westrip, 2010 ▸).

## supplementary crystallographic information

### 2-Oxo-N-(pyridin-4-yl)-2H-chromene-3-carboxamide Crystal data

**Table:**

C_15_H_10_N_2_O_3_	*Z* = 2
*M**_r_* = 266.25	*F*(000) = 276
Triclinic, *P*1	*D*_x_ = 1.494 Mg m^−^^3^
Hall symbol: -P 1	Melting point: 410 K
*a* = 5.731 (2) Å	Mo *K*α radiation, λ = 0.71073 Å
*b* = 7.668 (3) Å	Cell parameters from 2253 reflections
*c* = 13.911 (5) Å	θ = 0.2–29.0°
α = 103.199 (14)°	µ = 0.11 mm^−^^1^
β = 93.059 (11)°	*T* = 299 K
γ = 94.332 (10)°	Prism, colourless
*V* = 591.8 (4) Å^3^	0.32 × 0.27 × 0.24 mm

### 2-Oxo-N-(pyridin-4-yl)-2H-chromene-3-carboxamide Data collection

**Table:**

Bruker SMART APEXII CCD diffractometer	3181 independent reflections
Radiation source: fine-focus sealed tube	2263 reflections with *I* > 2σ(*I*)
Graphite monochromator	*R*_int_ = 0.034
Detector resolution: 1.7 pixels mm^-1^	θ_max_ = 29.7°, θ_min_ = 2.7°
φ and Ω scans	*h* = −7→7
Absorption correction: multi-scan (SADABS; Krause *et al.*, 2015)	*k* = −10→8
*T*_min_ = 0.964, *T*_max_ = 0.973	*l* = −19→19
8155 measured reflections	

### 2-Oxo-N-(pyridin-4-yl)-2H-chromene-3-carboxamide Refinement

**Table:**

Refinement on *F*^2^	Primary atom site location: structure-invariant direct methods
Least-squares matrix: full	Secondary atom site location: difference Fourier map
*R*[*F*^2^ > 2σ(*F*^2^)] = 0.071	Hydrogen site location: inferred from neighbouring sites
*wR*(*F*^2^) = 0.184	H-atom parameters constrained
*S* = 1.02	*w* = 1/[σ^2^(*F*_o_^2^) + (0.0775*P*)^2^ + 0.3022*P*] where *P* = (*F*_o_^2^ + 2*F*_c_^2^)/3
3181 reflections	(Δ/σ)_max_ < 0.001
181 parameters	Δρ_max_ = 0.35 e Å^−^^3^
0 restraints	Δρ_min_ = −0.22 e Å^−^^3^
0 constraints	

### 2-Oxo-N-(pyridin-4-yl)-2H-chromene-3-carboxamide Special details

**Table:**

Geometry. All esds (except the esd in the dihedral angle between two l.s. planes) are estimated using the full covariance matrix. The cell esds are taken into account individually in the estimation of esds in distances, angles and torsion angles; correlations between esds in cell parameters are only used when they are defined by crystal symmetry. An approximate (isotropic) treatment of cell esds is used for estimating esds involving l.s. planes.

### 2-Oxo-N-(pyridin-4-yl)-2H-chromene-3-carboxamide Fractional atomic coordinates and isotropic or equivalent isotropic displacement parameters (Å^2^)

**Table:**

	*x*	*y*	*z*	*U*_iso_*/*U*_eq_	
O1	0.6621 (2)	0.66045 (19)	0.30075 (10)	0.0408 (4)	
N1	0.5999 (3)	0.8748 (2)	0.60639 (12)	0.0391 (4)	
H1	0.709158	0.903908	0.571403	0.047*	
C1	0.6267 (3)	0.9567 (2)	0.70744 (14)	0.0343 (4)	
O2	0.2543 (3)	0.7009 (2)	0.59463 (11)	0.0530 (5)	
C2	0.8347 (4)	1.0609 (3)	0.74418 (16)	0.0429 (5)	
H2	0.951919	1.076508	0.702375	0.051*	
O3	0.8239 (3)	0.8350 (2)	0.43803 (12)	0.0546 (5)	
C3	0.8642 (4)	1.1408 (3)	0.84403 (17)	0.0489 (6)	
H3	1.003647	1.211559	0.867377	0.059*	
N2	0.7074 (4)	1.1241 (3)	0.90939 (14)	0.0500 (5)	
C5	0.5084 (4)	1.0255 (3)	0.87212 (16)	0.0463 (5)	
H5	0.394600	1.012612	0.915832	0.056*	
C6	0.4575 (4)	0.9411 (3)	0.77383 (15)	0.0407 (5)	
H6	0.313624	0.875356	0.752407	0.049*	
C7	0.4221 (3)	0.7544 (3)	0.55616 (15)	0.0368 (4)	
C8	0.4456 (3)	0.6886 (3)	0.44736 (14)	0.0339 (4)	
C9	0.2666 (3)	0.5796 (3)	0.39258 (14)	0.0357 (4)	
H9	0.133815	0.550586	0.423391	0.043*	
C10	0.2754 (3)	0.5076 (3)	0.28884 (14)	0.0343 (4)	
C11	0.4783 (3)	0.5494 (3)	0.24507 (14)	0.0354 (4)	
C12	0.6545 (4)	0.7357 (3)	0.39968 (14)	0.0374 (4)	
C13	0.0925 (4)	0.3980 (3)	0.22872 (16)	0.0430 (5)	
H13	−0.045401	0.368823	0.256137	0.052*	
C14	0.1153 (4)	0.3332 (3)	0.12939 (16)	0.0471 (5)	
H14	−0.007513	0.261687	0.089546	0.056*	
C15	0.3218 (4)	0.3747 (3)	0.08887 (16)	0.0486 (6)	
H15	0.337020	0.328822	0.021821	0.058*	
C16	0.5058 (4)	0.4830 (3)	0.14592 (15)	0.0443 (5)	
H16	0.644142	0.510211	0.118265	0.053*	

### 2-Oxo-N-(pyridin-4-yl)-2H-chromene-3-carboxamide Atomic displacement parameters (Å^2^)

**Table:**

	*U*^11^	*U*^22^	*U*^33^	*U*^12^	*U*^13^	*U*^23^
O1	0.0374 (8)	0.0506 (9)	0.0319 (7)	−0.0050 (6)	0.0080 (6)	0.0060 (6)
N1	0.0397 (9)	0.0443 (10)	0.0312 (8)	−0.0038 (7)	0.0077 (7)	0.0060 (7)
C1	0.0399 (10)	0.0314 (9)	0.0316 (9)	0.0028 (8)	0.0029 (8)	0.0071 (7)
O2	0.0484 (9)	0.0697 (11)	0.0352 (8)	−0.0153 (8)	0.0113 (7)	0.0051 (7)
C2	0.0406 (11)	0.0458 (12)	0.0414 (11)	−0.0025 (9)	0.0062 (9)	0.0099 (9)
O3	0.0417 (9)	0.0716 (11)	0.0425 (9)	−0.0166 (8)	0.0091 (7)	0.0029 (8)
C3	0.0470 (12)	0.0492 (13)	0.0440 (12)	−0.0108 (10)	−0.0022 (10)	0.0034 (10)
N2	0.0584 (12)	0.0505 (11)	0.0364 (10)	−0.0014 (9)	0.0012 (8)	0.0026 (8)
C5	0.0511 (13)	0.0494 (12)	0.0361 (11)	0.0007 (10)	0.0094 (9)	0.0051 (9)
C6	0.0395 (11)	0.0416 (11)	0.0371 (11)	−0.0039 (8)	0.0042 (8)	0.0035 (8)
C7	0.0386 (10)	0.0386 (10)	0.0326 (10)	0.0012 (8)	0.0043 (8)	0.0073 (8)
C8	0.0344 (10)	0.0371 (10)	0.0306 (9)	0.0011 (8)	0.0053 (7)	0.0088 (7)
C9	0.0353 (10)	0.0386 (10)	0.0337 (10)	0.0011 (8)	0.0059 (8)	0.0093 (8)
C10	0.0372 (10)	0.0338 (10)	0.0313 (10)	0.0033 (8)	0.0031 (8)	0.0065 (7)
C11	0.0384 (10)	0.0342 (10)	0.0339 (10)	0.0025 (8)	0.0044 (8)	0.0084 (8)
C12	0.0395 (10)	0.0386 (10)	0.0334 (10)	0.0006 (8)	0.0056 (8)	0.0073 (8)
C13	0.0411 (11)	0.0443 (12)	0.0412 (12)	−0.0013 (9)	0.0039 (9)	0.0062 (9)
C14	0.0520 (13)	0.0454 (12)	0.0389 (11)	−0.0011 (10)	−0.0027 (10)	0.0028 (9)
C15	0.0678 (15)	0.0482 (13)	0.0284 (10)	0.0098 (11)	0.0049 (10)	0.0045 (9)
C16	0.0479 (12)	0.0509 (13)	0.0356 (11)	0.0037 (10)	0.0118 (9)	0.0116 (9)

### 2-Oxo-N-(pyridin-4-yl)-2H-chromene-3-carboxamide Geometric parameters (Å, º)

**Table:**

O1—C12	1.370 (2)	C5—H5	0.9300
O1—C11	1.382 (2)	C6—H6	0.9300
N1—C7	1.366 (3)	C7—C8	1.499 (3)
N1—C1	1.398 (2)	C8—C9	1.352 (3)
N1—H1	0.8600	C8—C12	1.461 (3)
C1—C2	1.388 (3)	C9—C10	1.428 (3)
C1—C6	1.391 (3)	C9—H9	0.9300
O2—O2	0.000 (5)	C10—C11	1.391 (3)
O2—O2	0.000 (5)	C10—C13	1.399 (3)
O2—C7	1.215 (2)	C11—C16	1.379 (3)
C2—C3	1.380 (3)	C13—C14	1.374 (3)
C2—H2	0.9300	C13—H13	0.9300
O3—O3	0.000 (5)	C14—C15	1.384 (3)
O3—C12	1.206 (2)	C14—H14	0.9300
C3—N2	1.332 (3)	C15—C16	1.381 (3)
C3—H3	0.9300	C15—H15	0.9300
N2—C5	1.330 (3)	C16—H16	0.9300
C5—C6	1.378 (3)		
			
C12—O1—C11	122.70 (15)	C9—C8—C7	118.04 (17)
C7—N1—C1	128.19 (17)	C12—C8—C7	122.45 (17)
C7—N1—H1	115.9	C8—C9—C10	121.95 (18)
C1—N1—H1	115.9	C8—C9—H9	119.0
C2—C1—C6	117.69 (18)	C10—C9—H9	119.0
C2—C1—N1	118.11 (18)	C11—C10—C13	118.14 (18)
C6—C1—N1	124.20 (18)	C11—C10—C9	117.87 (18)
C3—C2—C1	118.6 (2)	C13—C10—C9	123.99 (18)
C3—C2—H2	120.7	C16—C11—O1	117.38 (17)
C1—C2—H2	120.7	C16—C11—C10	122.19 (19)
N2—C3—C2	124.8 (2)	O1—C11—C10	120.43 (17)
N2—C3—H3	117.6	O3—C12—O1	115.52 (17)
C2—C3—H3	117.6	O3—C12—O1	115.52 (17)
C5—N2—C3	115.42 (19)	O3—C12—C8	127.00 (19)
N2—C5—C6	125.1 (2)	O3—C12—C8	127.00 (19)
N2—C5—H5	117.4	O1—C12—C8	117.48 (17)
C6—C5—H5	117.4	C14—C13—C10	120.4 (2)
C5—C6—C1	118.3 (2)	C14—C13—H13	119.8
C5—C6—H6	120.8	C10—C13—H13	119.8
C1—C6—H6	120.8	C13—C14—C15	119.8 (2)
O2—C7—N1	123.95 (19)	C13—C14—H14	120.1
O2—C7—N1	123.95 (19)	C15—C14—H14	120.1
O2—C7—N1	123.95 (19)	C16—C15—C14	121.4 (2)
O2—C7—C8	120.60 (18)	C16—C15—H15	119.3
O2—C7—C8	120.60 (18)	C14—C15—H15	119.3
O2—C7—C8	120.60 (18)	C11—C16—C15	118.08 (19)
N1—C7—C8	115.45 (16)	C11—C16—H16	121.0
C9—C8—C12	119.50 (18)	C15—C16—H16	121.0
			
C7—N1—C1—C2	173.33 (19)	C8—C9—C10—C11	1.6 (3)
C7—N1—C1—C6	−6.9 (3)	C8—C9—C10—C13	−178.3 (2)
C6—C1—C2—C3	0.9 (3)	C12—O1—C11—C16	179.46 (18)
N1—C1—C2—C3	−179.3 (2)	C12—O1—C11—C10	−0.7 (3)
C1—C2—C3—N2	0.8 (4)	C13—C10—C11—C16	−1.7 (3)
C2—C3—N2—C5	−1.7 (4)	C9—C10—C11—C16	178.44 (19)
C3—N2—C5—C6	0.8 (4)	C13—C10—C11—O1	178.54 (18)
N2—C5—C6—C1	0.9 (4)	C9—C10—C11—O1	−1.4 (3)
C2—C1—C6—C5	−1.7 (3)	C11—O1—C12—O3	−177.37 (18)
N1—C1—C6—C5	178.55 (19)	C11—O1—C12—O3	−177.37 (18)
C1—N1—C7—O2	0.6 (3)	C11—O1—C12—C8	2.5 (3)
C1—N1—C7—O2	0.6 (3)	C9—C8—C12—O3	177.6 (2)
C1—N1—C7—O2	0.6 (3)	C7—C8—C12—O3	−3.4 (3)
C1—N1—C7—C8	−179.17 (18)	C9—C8—C12—O3	177.6 (2)
O2—C7—C8—C9	5.0 (3)	C7—C8—C12—O3	−3.4 (3)
O2—C7—C8—C9	5.0 (3)	C9—C8—C12—O1	−2.3 (3)
O2—C7—C8—C9	5.0 (3)	C7—C8—C12—O1	176.68 (17)
N1—C7—C8—C9	−175.21 (18)	C11—C10—C13—C14	0.5 (3)
O2—C7—C8—C12	−174.0 (2)	C9—C10—C13—C14	−179.6 (2)
O2—C7—C8—C12	−174.0 (2)	C10—C13—C14—C15	0.8 (3)
O2—C7—C8—C12	−174.0 (2)	C13—C14—C15—C16	−1.0 (4)
N1—C7—C8—C12	5.8 (3)	O1—C11—C16—C15	−178.72 (18)
C12—C8—C9—C10	0.3 (3)	C10—C11—C16—C15	1.5 (3)
C7—C8—C9—C10	−178.74 (17)	C14—C15—C16—C11	−0.1 (3)

### 2-Oxo-N-(pyridin-4-yl)-2H-chromene-3-carboxamide Hydrogen-bond geometry (Å, º)

**Table:**

*D*—H···*A*	*D*—H	H···*A*	*D*···*A*	*D*—H···*A*
N1—H1···O3	0.86	1.97	2.698 (2)	141
C6—H6···O2	0.93	2.29	2.869 (3)	120
C9—H9···O2	0.93	2.44	2.758 (3)	100
C2—H2···O3^i^	0.93	2.58	3.480 (3)	162
C13—H13···O2^ii^	0.93	2.59	3.422 (3)	149

Symmetry codes: (i) −*x*+2, −*y*+2, −*z*+1; (ii) −*x*, −*y*+1, −*z*+1.
